# Examination of *Sarcocystis* spp. of giant snakes from Australia and Southeast Asia confirms presence of a known pathogen – *Sarcocystis nesbitti*

**DOI:** 10.1371/journal.pone.0187984

**Published:** 2017-11-13

**Authors:** Marion Wassermann, Lisa Raisch, Jessica Ann Lyons, Daniel James Deans Natusch, Sarah Richter, Mareike Wirth, Piyarat Preeprem, Yuvaluk Khoprasert, Sulaiman Ginting, Ute Mackenstedt, Thomas Jäkel

**Affiliations:** 1 Department of Zoology, Division of Parasitology, University of Hohenheim, Stuttgart, Germany; 2 Resource Evaluation and Development, Bamaga, Queensland, Australia; 3 School of Life and Environmental Sciences, University of Sydney, Sydney, NSW, Australia; 4 Plant Protection Research and Development Office, Department of Agriculture, Bangkok, Thailand; 5 Islamic University of North Sumatra, Medan, Indonesia; National Cheng Kung University, TAIWAN

## Abstract

We examined *Sarcocystis* spp. in giant snakes from the Indo-Australian Archipelago and Australia using a combination of morphological (size of sporocyst) and molecular analyses. We amplified by PCR nuclear 18S rDNA from single sporocysts in order to detect mixed infections and unequivocally assign the retrieved sequences to the corresponding parasite stage. *Sarcocystis* infection was generally high across the study area, with 78 (68%) of 115 examined pythons being infected by one or more *Sarcocystis* spp. Among 18 randomly chosen, sporocyst-positive samples (11 from Southeast Asia, 7 from Northern Australia) the only *Sarcocystis* species detected in Southeast Asian snakes was *S*. *singaporensis* (in reticulated pythons), which was absent from all Australian samples. We distinguished three different *Sarcocystis* spp. in the Australian sample set; two were excreted by scrub pythons and one by the spotted python. The sequence of the latter is an undescribed species phylogenetically related to *S*. *lacertae*. Of the two *Sarcocystis* species found in scrub pythons, one showed an 18S rRNA gene sequence similar to *S*. *zamani*, which is described from Australia for the first time. The second sequence was identical/similar to that of *S*. *nesbitti*, a known human pathogen that was held responsible for outbreaks of disease among tourists in Malaysia. The potential presence of *S*. *nesbitti* in Australia challenges the current hypothesis of a snake-primate life cycle, and would have implications for human health in the region. Further molecular and biological characterizations are required to confirm species identity and determine whether or not the Australian isolate has the same zoonotic potential as its Malaysian counterpart. Finally, the absence of *S*. *nesbitti* in samples from reticulated pythons (which were reported to be definitive hosts), coupled with our phylogenetic analyses, suggest that alternative snake hosts may be responsible for transmitting this parasite in Malaysia.

## Introduction

The genus *Sarcocystis* comprises a group of more than 200 species of protozoan parasites that infect mammals, reptiles, birds, and fish. *Sarcocystis* spp. usually require two hosts to complete their life cycle [1,2]. Typically, a carnivorous definitive host preys on an infected omnivorous or herbivorous intermediate host, and the parasite undergoes sexual development within the definitive host’s intestine [3]. *Sarcocystis* parasites using snakes as definitive hosts are of particular interest for several reasons. For example, a high infectious dose of *S*. *singaporensis* is pathogenic to its intermediate host, and has been used effectively as a biological rodenticide [4]. In this species, natural intermediate hosts include rats of the genera *Rattus* and *Bandicota*, while the giant reticulated python (*Malayopython reticulatus*) serves as the definitive host [5–8].

Other snake-host *Sarcocystis* spp. also are presumed to be pathogenic to humans. Recent outbreaks of disease among tourists in Malaysia were attributed to *S*. *nesbitti*, which was identified as the causative agent based on molecular identification of its 18S rRNA gene isolated from muscle cysts (sarcocysts) of patients suffering from acute infection accompanied by myositis [9–11]. An earlier record of *Sarcocystis*-infection and disease among US military personnel in Malaysia in 1993 could have been caused by the same pathogen, although *S*. *nesbitti* was not implicated at that time [12]. When searching for the definitive host of *S*. *nesbitti*, phylogenetic analyses of its 18S rRNA gene predicted snakes as likely candidates, since the sequence clustered with those from other snake-specific *Sarcocystis* spp. [13]. The studies that followed this discovery examined a range of snake species, in Malaysia and elsewhere [14,15]. Based on molecular analyses of total DNA extracted from faecal samples using the 18S rDNA sequence as a PCR probe, *S*. *nesbitti* was reported to be present in the reticulated python and a monocled cobra (*Naja kaouthia*) [14,16]. The authors concluded that the snakes were infected with *S*. *nesbitti* and served as definitive hosts. However, in none of the faecal samples were sporocysts or oocysts–evidence of infection–identified or examined. Thus, in our view the identity of the definitive host of *S*. *nesbitti* remains uncertain.

The findings above implicate snakes as potential carriers of human pathogens, prompting the need for more thorough knowledge of the distribution and host-parasite relationships of *Sarcocystis* spp. in snakes. In this study, we screened extensively for *Sarcocystis* spp. within pythons from Southeast Asia, New Guinea (i.e., green tree pythons from Indonesian Papua), and Australia, by detecting sporocysts in faecal samples through a flotation technique and, subsequently, employing a PCR protocol that allows amplification of the 18S rRNA gene from a single sporocyst (the stage released by the definitive host; [17]). This molecular approach directly targets the most relevant parasite stage, without requiring animal experimentation typical of other *Sarcocystis* life cycle and taxonomic studies. Our results expand our knowledge of *Sarcocystis* spp. in the region and identify a definitive host of *S*. *nesbitti*.

## Material and methods

### Ethics statement

Our data were gathered from snakes brought to animal rescue centers and public research institutions, kept by local traders, or captured by ourselves in the wild. We stress that no snakes were harmed for the purpose of our study. In case of direct capture, we released all animals unharmed at the site of collection after examination. Wild-caught reticulated pythons and other python species from Southeast Asia were examined with permission of Jong’s Crocodile Farm & Zoo, Kuching (Malaysia), the Agricultural Zoology Research Station, Department of Agriculture, Ministry of Agriculture and Cooperatives (Thailand), the Vocational Education and Development Center for Agriculture (VEDCA), Ministry of Education, Cianjur (Indonesia), the Institute for Agricultural Technology of North Sumatra (BPTP-SU) in Medan under the Ministry of Agriculture and Forestry (Indonesia), and the Bureau of Plant Industry (BPI) and Philippine Rice Research Institute of the Department of Agriculture (Philippines). Permission was granted on basis of bilateral collaboration agreements between the above institutions and German International Cooperation (GIZ; Project No. 2002.2156.4) under commission of the German Federal Ministry of Economic Cooperation and Development (BMZ). Sampling in Australia was permitted by the Queensland Department of Environment and Heritage Protection (permit No: WISP12944313) and the University of Sydney Animal Ethics Committee (protocol No: L04/3-2013/3/5969).

### Origin of parasite isolates and sampling procedures

We originally isolated oocysts/sporocysts of *Sarcocystis* spp. from Southeast Asia from faeces collected from adult wild-caught pythons brought to research institutes and animal rescue centres (e.g., Thailand, Borneo) or from animal traders (e.g., Sumatra, from python skin processing facilities). We collected samples from 75 reticulated pythons (*Malayophyton reticulatus*), five green tree pythons (*Morelia viridis*) and three Burmese pythons (*Python bivittatus*). Only scats that had been freshly discharged by pythons and could be unequivocally assigned to a specific individual were considered. Scats were stored in sterile plastic bags in the refrigerator at 4°C until further examination (usually no longer than 3 months).

Australian samples were collected from wild pythons captured by hand near the town of Bamaga at the northern tip of Cape York Peninsula, Queensland, Australia. We retrieved faecal samples by gently palpating the snake’s hind-gut until defecation occurred. Faeces were kept in sterile plastic vials in the refrigerator until further use. We released all animals unharmed at the site of capture after examination. In total, we obtained faecal material from 23 scrub pythons (*Simalia amethistina*), six spotted pythons (*Antaresia maculosa*) and three black-headed pythons (*Aspidites melanocephalus*).

### Screening for oocysts/sporocysts of *Sarcocystis* spp. in faecal samples

Oocysts/sporocysts were extracted from faecal pellets and concentrated using the standard zinc sulphate flotation technique (33%, w/v, zinc sulphate in distilled water). Briefly, one gram of faecal sample was emulsified in a porcelain beaker in 15–16 ml zinc sulphate solution and the suspension passed through brass wire into a 12.5 ml glass centrifuge tube. Each centrifuge tube was filled until a reverse meniscus was formed. Then, a glass coverslip was gently added to the top of the meniscus and positioned so it would not interfere with the centrifuge bucket. Using a swing-out rotor we centrifuged the samples with coverslips at 1,600 *g* for 2.5 min without applying a brake. We then lifted the coverslips and placed the adherent concentrates onto glass slides for examination under the microscope. All samples were also screened by direct (wet) smears under a microscope at 400-fold magnification. Length and diameter of sporocysts were measured using a calibrated microscope.

### Purification of oocysts/sporocysts in preparation for PCR

Our main subject of examination was the sporocyst; the stage of the genus *Sarcocystis* that develops in the intestine of the definitive host. Although development of sporocysts (sporulation) takes place inside a typical coccidian oocyst, the thin wall of the latter can rupture easily, releasing two sporocysts once in the lumen of the host’s gut [17]. Thus, oocysts usually occur shortly after infection, while only sporocysts may be seen later on. We developed a PCR approach that allowed amplification of the 18S rRNA gene from a single sporocyst. This had the advantage that multiple infections with different *Sarcocystis* spp. could be distinguished. Furthermore, we suspected that if total DNA was extracted from scats, contamination with *Sarcocystis*-stages in prey tissue might occur, since rDNA molecules appear sufficiently stable to be detectable by environmental DNA surveys (e.g., [18]). According to experiences of one of the authors, partly digested prey tissue can occur in scats of giant snakes, particularly if bigger prey items are swallowed (T. Jäkel, unpubl. observations).

Oocysts/sporocysts of *Sarcocystis*-positive samples were purified via zinc chloride flotation. For this purpose, we diluted 1 or 2 cm^3^ of faecal material with 10 ml phosphate-buffered saline (PBS) containing 0.3% Tween 20, centrifuged for 10 min at 1600 *g*, and discarded the supernatant. The pellet was re-suspended in 15 ml zinc chloride solution with a specific density of 1.45 g per cm^3^ and centrifuged for 30 min at 400 *g*. Then, all of the supernatant was passed through a 25 μm mesh, diluted with tap water (1:10), centrifuged for 10 min at 1600 *g* and the supernatant discarded. The pellet was re-suspended in 1 ml distilled water and stored at 4°C until further use.

In preparation for PCR analysis, single sporocysts were aspirated with a pipette under an inverted microscope, transferred to 0.2 ml reaction tubes containing 5 μl of 0.02 M NaOH, and subsequently lysed at 95°C for 15 min. The lysate was used directly as template for the following PCRs.

### PCR and sequencing of the 18S rRNA gene of single sporocysts

For each sample, 30 sporocysts were randomly chosen and examined by PCR. Some samples showed no or only few positive reactions. In those cases, the number of sporocysts chosen for examination was increased up to 100.

Because single sporocysts only contain minute amounts of DNA, we chose a nested PCR approach. The 18S rRNA gene was amplified in two overlapping fragments. The first PCR step was performed in a volume of 25 μl, containing 10 mM Tris-HCl (pH 8.3), 50 mM KCl, 2 mM MgCl_2_, 200 μM of each dNTP, 6.25 pmol of each external primer, and 0.625 U Ampli-Taq Polymerase (Applied Biosystems) and 1.5 μl of the sporocyst lysate. For the nested PCRs (the second step) volumes were increased to 50 μl, containing 10 mM Tris-HCl (pH 8.3), 50 mM KCl, 2 mM MgCl_2_, 200 μM of each dNTP, 12.5 pmol of each internal primer and 1.25 U Ampli-Taq Polymerase and 3 μl of the first PCR product. The obtained fragment of the front part was approximately 1020 base pairs (bp) long and the back part 900 bp. We designed the primers (Table 1) from the conserved regions of the 18S rRNA gene of *S*. *hirsuta* (AF017122; [19]) and *S*. *singaporensis* (AF434054; [20]). All amplification reactions started with an initial denaturation at 95°C for 5 min, followed by 35 cycles of denaturation for 30 s at 95°C, annealing for 30 s at 50°C, elongation for 60 s at 72°C, and a final elongation step for 5 min at 72°C. The amplicons were sequenced by GATC Biotech AG (Konstanz, Germany). To join the two fragments, the sequences were aligned, the overlapping region identified and concatenated to a single sequence. The two fragments had to be obtained from a single sporocyst. All concatenated sequences identified in this study were regarded as valid when at least two concatenated sequences from different sporocysts showed 100% identity. Joint sequences were submitted to GenBank under the indicated accession numbers. Using the NCBI BLAST online tool the obtained sequences were compared with already existing data in the GenBank.

**Table 1:** Primers used for amplification of the 18S rRNA gene of *Sarcocystis* spp.

18S rRNA fragment	Primers for the first PCR (5’-3’)	Primers for the nested PCR (5’-3’)
Front part	F: TATGCTTGTCTTAAAGATTAAGCC	F: AGCCATGCATGTCTAAGTATAAGC
	R: ACTTTGATTTCTCATAAGGTGC	R: GACTACGACGGTATCTTATCGTC
Back part	F: AGTAATGATTAATAGGGACAGTTG	F: GCATTCGTATTTAACTGTCAGAGG
	R: GTGAATGATCCTTCCGCAGGTTCA	R: CTACGGAAACCTTGTTACGACTTC

### Phylogenetic analyses

The 18S rRNA gene sequences of the *Sarcocystis* isolates under investigation (including the variable regions of the gene; [20]) were aligned with published Apicomplexan species available at GenBank [21] using the multiple sequence alignment algorithm of the ‘R-Coffee’ web server, which takes into account predicted secondary structures of RNA [22]. In the case of *S*. *zamani*, where the 18S rDNA sequence had been deposited in conjunction with neighbouring genes (KU2445241), we excised the relevant part after alignment with the homologous sequences of *Toxoplasma gondii* (M97703) and *S*. *singaporensis* (Sin-T; see results). The appropriate model for nucleotide substitution rates was determined using the jModel Test software version 2.0 [23]. Evolutionary distances were computed using the Maximum Composite Likelihood method with Gamma-distributed substitutions (G) and variation among lineages (as observed for the coccidia; [24]) under the Minimum Evolution (ME) algorithm, or the Tamura-Nei-93 model with G and invariant sites (I) under the Maximum Likelihood (ML) algorithm. Substitution rate variation among sites was estimated by ML over five Gamma categories and the number of sites to be analysed. Site coverage for ME and ML was between 79% and 86%; that is, fewer than 14%–21% alignment gaps, missing data, and ambiguous bases were allowed at any position. There were a total of 1544–1565 positions in the final dataset. All phylogenetic trees were tested by 1000 bootstrap iterations. We conducted all analyses using the MEGA 6 software package [25].

## Results

### Prevalence of *Sarcocystis* spp. in faecal samples from pythons and size classes of sporocysts

In total, we examined 115 faecal samples from Southeast Asian and Australian pythons for *Sarcocystis* infection. Using the zinc sulphate flotation technique, *Sarcocystis* sporocysts could be detected in 78 samples (Table 2), which is an overall prevalence of 68%. Sporocysts were also readily detectable in 66 (85%) direct smears of the flotation-positive samples. However, prevalence varied between python species and geographic origin. The lowest infection rate (17%) was observed in the Australian spotted python (*Antaresia maculosa*) and the highest prevalence (100%) in reticulated pythons from Sumatra.

**Table 2:** Sites of origin, python species, prevalence of infection with *Sarcocystis* spp., and size classes of sporocysts detected in faecal samples from pythons of Southeast Asia, New Guinea, and Australia.

Geographic origin	Geo-reference	Python species	No. examined / No. infected (No. mixed infections)	Prevalence of infection (%)	Sporocyst size class^a^
Thailand (Bangkok Metropolis, Nakorn Pathom, Surat Thani)	13°50'49" N 100°34'18" E; 13°52'12" N 100°04'47" E; 09°09'14"N 99°17'41"E	*Malayopython reticulatus*	22 / 20 (3)	91	class 1 (20), class 2 (3)
Indonesia, Sumatra (Medan)	03°29'24" N 98°37'30" E	dto.	18 / 18 (2)	100	class 1 (18), class 2 (2)
Indonesia, Sulawesi (Makkassar)	05°11'50" S 119°39'22" E	dto.	7 / 3	43	class 1
Indonesia, Java (Cianjur)	06°47'25" S 107°09'37" E	dto.	11 / 6	55	class 1
Malaysia, peninsular (Selangor)	03°05'59" N 101°30'34" E	dto.	2 / 1	50	class 1
Malaysia, Borneo (Kuching)	01°21'43" N 110°24'52" E	dto.	3 / 1	33	class 1
Singapore	01°24'20" N 103°47'24" E	dto.	4 / 2 (1)	50	class 1 (2), class 2 (1)
Philippines, Luzon (Bulacan, Cavite, Bicol)	14°56'43" N 121°06'40" E; 14°13'47" N 120°51'59" E; 13°25'05" N 123°26'16" E	dto.	5 / 4	80	class 1
Philippines, Mindanao (Agusan del Norte)	08°57'13" N 125°37'29" E	dto.	3 / 2 (1)	67	class 1 (2), class 2 (1)
Indonesia, Papua (Aru, Jayapura)	06°11'02" S 134°20'58" E; 02°33'37" S 140°38'24" E	*Morelia viridis*	5 / 4	80	class 1
Thailand (Bangkok Metropolis)	13°51'59" N 100°40'20" E	*Python bivittatus*	3 / 0	0	none
Australia, Cape York (Bamaga)	10°50'01" S 142°28'52" E	*Antharesia maculosa*	6 / 1	17	class 3
Australia, Cape York (Bamaga)	10°50'01" S 142°28'52" E	*Aspidites melanocephalus*	3 / 1	33	Not available^b^
Australia, Cape York (Bamaga)	10°50'01" S 142°28'52" E	*Simalia amethistina*	23 / 15 (3)	65	class 1 (15), class 4 (3)

^a^Mean sizes of sporocysts in the samples were compared with reference values: reference values for size class 1 (= *S*. *singaporensis*-like sporocysts) and size class 2 (= *S*. *zamani*-like sporocysts) were 10.2 (±0.8; ±S.D.) x 7.9 (±0.7) μm (n = 350) and 9.7 (±0.6) x 7.6 (±0.2) μm (n = 50), respectively. These measurements were based on experimental infections of *Malayopython reticulatus* (reticulated python) with isolates from Thailand [26,27]. Sporocysts of even smaller size have been described for *S*. *zamani* [27]. Size classes 3 and 4 are defined in the text.

^b^Sporocysts of *Sarcocystis* sp. in *Aspidites melanocephalus* (black-headed python) were larger than any of the size classes. However, concentration of sporocysts was too low to measure sufficient numbers for reliable average values.

Based on our measurements of sporocyst dimensions and comparisons with previously published work on *Sarcocystis* spp. of pythons, we could distinguish four classes of sporocyst sizes among all samples (Table 2). Measurements from Southeast Asia fell into two size categories. Class 1 (*S*. *singaporensis*) and class 2 (*S*. *zamani*) were used as reference values and related to previous work that could unequivocally assign mean sporocyst sizes to these two species through experimental infections of reticulated pythons [26,27]. Means and variances of reference values for class 1 and 2 are provided in footnote ‘a’ of Table 2. Sporocysts of class 2 were significantly smaller in length and diameter than those of class 1 (T-test: length, *P* ≤ 0.001, *t* = -4.250, *d*.*f*. = 398; diameter, *P* = 0.003, *t* = -3.010, *d*.*f*. = 398). For instance, typical mean sizes of sporocysts from reticulated pythons in Bangkok and Sulawesi were 10.0 (± 0.5; ±S.D.) x 7.5 (±0.3) μm (n = 30) and 10.4 (± 0.4) x 8.1 (±0.4) μm (n = 20), respectively, which was not statistically different to the class 1 reference values. Hence, they were categorised as class 1. Molecular analysis of randomly chosen subsets of samples confirmed that size class 1 referred to *S*. *singaporensis* in Southeast Asia (Table 3; e.g. see samples 9 and 11).

**Table 3:** Geographic origin, snake hosts, sporocyst size classes, and partial 18S rRNA gene sequences of *Sarcocystis* spp. identified in faecal samples from pythons of Southeast Asia and Australia.

Sample No.	Geographic origin	Python species^a^	Sporocyst size class	Species of *Sarcocystis*	Size (nt)	Sequence code	Genbank Accession No.
1	Malaysia, Borneo (Kuching)	*M*.*r*.	class 1	*S*. *singaporensis*	816		n.s.^b^
2	Philippines, Luzon	*M*.*r*.	class 1	*S*. *singaporensis*	837		n.s.
3	Philippines, Luzon	*M*.*r*.	n.d.	*S*. *singaporensis*	429		n.s.
4	Philippines, Luzon	*M*.*r*.	n.d.	*S*. *singaporensis*	899		n.s.
5	Philippines, Luzon (Bicol)	*M*.*r*.	n.d.	*S*. *singaporensis*	1013		n.s.
6	Philippines, Mindanao (Agusan del Norte)	*M*.*r*.	class 1	*S*. *singaporensis*	422		n.s.
7	Indonesia, Java (Cianjur)	*M*.*r*.	n.d.	*S*. *singaporensis*	874		n.s.
8	Indonesia, Java (Cianjur)	*M*.*r*.	class 1	*S*. *singaporensis*	896		n.s.
9	Indonesia, Sulawesi (Makkassar)	*M*.*r*.	class 1	*S*. *singaporensis*	765		n.s.
10	Indonesia, Sumatra (Medan)	*M*.*r*.	class 1	*S*. *singaporensis*	951		n.s.
11	Thailand (Bangkok)	*M*.*r*.	class 1	*S*. *singaporensis*	1729	Sin-T	KY513624
12	Australia, Cape York (Bamaga)	*A*.*m*.	class 3	*S*. sp.2	1614	S.sp.2/28	KY513629
13	Australia, Cape York (Bamaga)	*S*.*a*.	n.d.	*S*. sp.1	1641	S.sp.1-1/2	KY513625
14	Australia, Cape York (Bamaga)	*S*.*a*.	n.d.	*S*. sp.1	1646	S.sp.1-3/15	KY513628
15	Australia, Cape York (Bamaga)	*S*.*a*.	class 1	*S*. sp.1	1677		n.s.
16	Australia, Cape York (Bamaga)	*S*.*a*.	n.d.	*S*. sp.1	777		n.s.
17	Australia, Cape York (Bamaga)	*S*.*a*.	class 1	*S*. sp.1	1641		n.s.
18	Australia, Cape York (Bamaga)	*S*.*a*.	class 1 class 4	*S*. sp.1 *S*. *nesbitti*	1641 1721	S.sp.1-2/6 S.nesb./6	KY513627 KY513626

^a^*A*.*m*. = *Antaresia maculosa*; *M*.*r*. = *Malayopython reticulatus*; *S*.*a*. = *Simalia amethistina*

^b^n.s. = not submitted, n.d. = not determined

Three sporocyst size classes were present in the Australian samples (Table 2). Sporocysts isolated from the scrub python (designated here as *Sarcocystis* sp.1) were similar in size to *S*. *singaporensis* (class 1) but constituted a genetically different species (see below). Sporocysts isolated from one spotted python measured 11.2 (± 0.5, ±S.D.) x 8.9 (±0.6) μm (n = 30), which was significantly longer and wider than the class 1 reference values (T-test: length, *P* ≤ 0.001, *t* = 6.730, d.f. = 378; diameter, *P* ≤ 0.001, *t* = 7.587, d.f. = 378). Therefore, we defined sporocysts of this sample as size class 3. A unique population of sporocysts, here designated as class 4 (11.2 [± 0.2] x 8.0 [± 0.4] μm, n = 30), was observed among three mixed infections with *Sarcocystis* sp.1 in the scrub python. Sporocysts of class 4 were significantly longer than class 1 (T-test: *P* ≤ 0.001, *t* = 6.821, d.f. = 378) but similar in diameter. They were similar in length, but smaller in diameter if compared to class 3 (T-test: diameter, *P* ≤ 0.001, *t* = -6.836, d.f. = 58).

### Molecular and phylogenetic analysis of 18S rDNA from *Sarcocystis* sporocysts

Out of the 78 *Sarcocystis*-positive faecal samples, 18 (11 from Southeast Asia, 7 from Northern Australia) were randomly chosen for each snake species at each location and subjected to further molecular analysis. We did not include samples from the green tree python (*Morelia viridis*) of Papua (Indonesia) here, because molecular investigations are still ongoing. In total, we identified four different *Sarcocystis* spp., one in Southeast Asia and three in the Australian sample set (Table 3).

**Fig 1:**
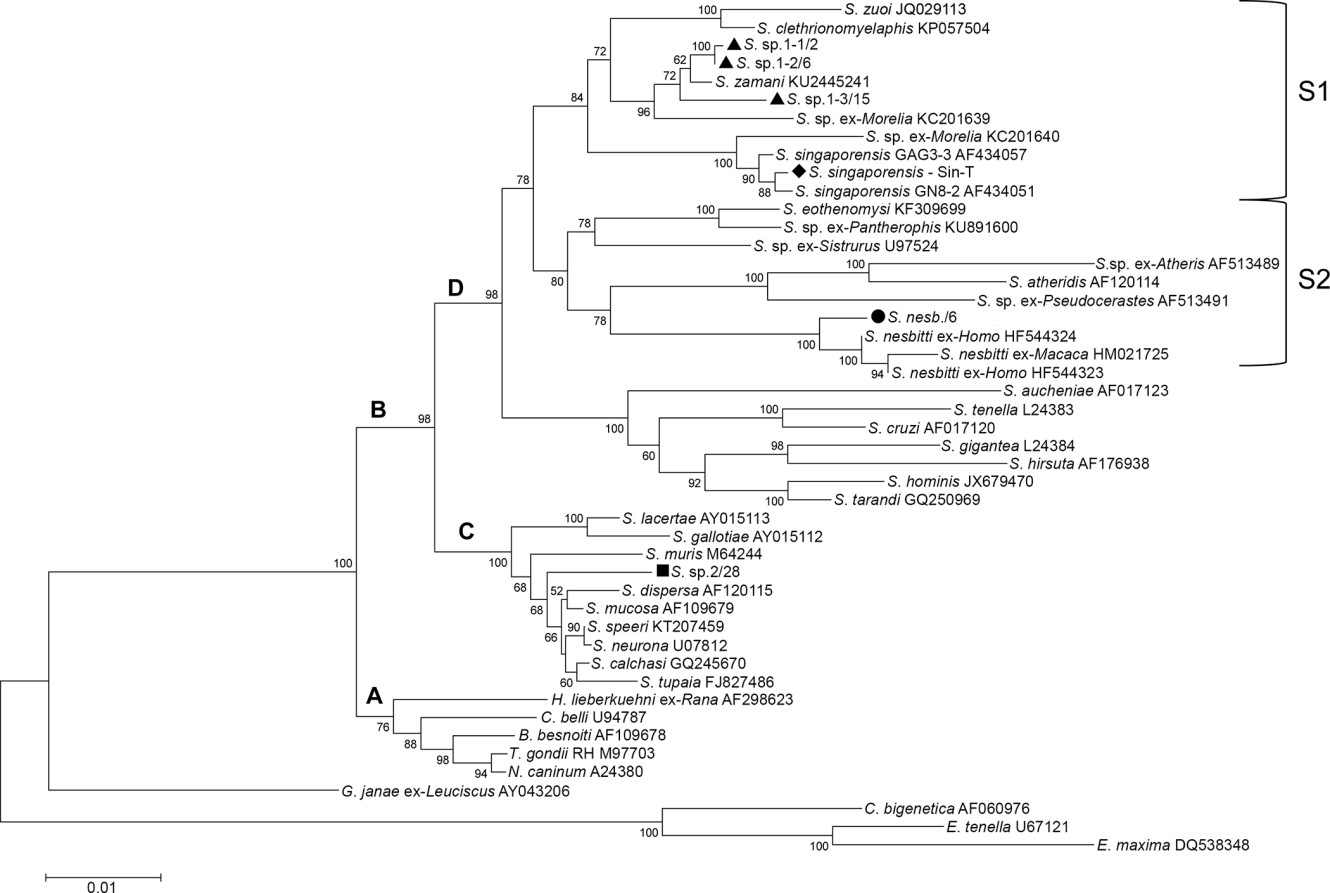
Phylogram of selected taxa of the Sarcocystidae including the new *Sarcocystis* isolates and species examined in this study (black symbols). Selected taxa of the Eimeriidae and the fish-host species *Goussia janae* served as root. Branches A and B indicate the subfamilies Toxoplasmatinae and Sarcocystinae, respectively. In addition to snake-host taxa, branch D includes *Sarcocystis* spp. of ruminating mammals as intermediate hosts. The tree was reconstructed by Minimum Evolution (ME) algorithm with 1557 sites of the 18S rRNA gene under analysis. Bootstrap values ≥ 50% of 1000 iterations are indicated. See further explanations in the text. Key to generic name abbreviations as follows: *B* = *Besnoitia*, *C* = *Cystoisospora*, *E* = *Eimeria*, *G* = *Goussia*, *H* = *Hyaloklossia*, *N* = *Neospora*, *S* = *Sarcocystis*, *T* = *Toxoplasma*.

The only Southeast Asian *Sarcocystis* sp. detected by PCR was *S*. *singaporensis* from the reticulated python (Thailand isolate Sin-T: Accession No. KY513624, Table 3, Fig 1). Although there were two size classes present in mixed infections as observed under the microscope, the larger *S*. *singaporensis*-like (class 1) and the smaller *S*. *zamani*-like (class 2) sporocysts (Table 2), we could not amplify 18S rDNA of *S*. *zamani*, because only one mixed infection was chosen for analysis and concentration of *S*. *zamani*-like sporocysts was too low (sample 6 from Mindanao). We obtained partial 18S rDNA sequences of *S*. *singaporensis* from a wide range of locations in Southeast Asia (Table 3), which were all highly similar (99%-100%) to sample 11 from Thailand (Sin-T) in pair-wise comparisons using the posterior fragment of the 18S rRNA gene.

**Fig 2:**
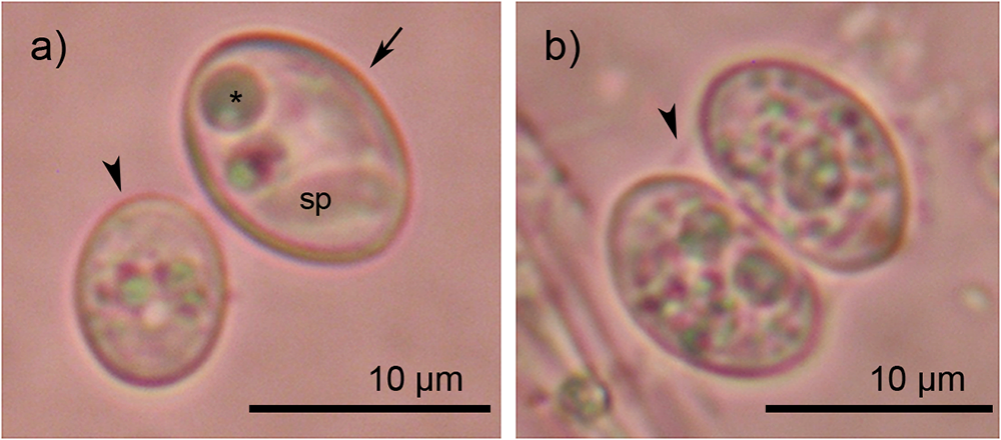
**a) Sporocysts of *Sarcocystis* cf. *nesbitti* (arrow) and *Sarcocystis* sp.1 (arrowhead, tentatively *S*. *zamani*) from the Australian scrub python.** Asterisk indicates residual body, sp = sporozoite; **b) Oocyst of *S*. cf. *nesbitti*.** Arrowhead indicates the thin oocyst wall.

We observed three different *Sarcocystis* species in the samples from Northern Australia. Molecular level differences corresponded closely with the different size classes of sporocysts. For instance, sporocysts of size class 3 from the spotted python (*A*. *maculosa*) revealed an 18S rDNA sequence of a so far unknown species, which we designate here as *Sarcocystis* sp.2 (Table 3, KY513629). Our phylogenetic analyses placed this species close to *S*. *lacertae* (branch C, Fig 1), which cycles between snakes and lizards [28]. The two other *Sarcocystis* spp. were both found in scrub pythons (*Simalia amethistina*). Interestingly, molecular analysis of class 1 sporocysts (Fig 2A) revealed that these sporocysts contained an 18S rDNA sequence that matched up to 99% with an isolate of *S*. *zamani* from a bandicoot rat in Thailand (clade S1 in Fig 1; [29]). Although this indicates that the Australian isolate could be *S*. *zamani*, we address this species here as *Sarcocystis* sp.1 (Table 3), because mean sizes of sporocysts were significantly larger than the reference values of class 2. There existed three different molecular variants of *Sarcocystis* sp.1 (Table 3). Pair-wise comparison of sequences S.sp.1-1/2 (KY513625) and S.sp.1-2/6 (KY513627) revealed only two nucleotide changes, whereas sequence S.sp.1-3/15 (KY513628) exhibited 34 changes of base pairs in comparison to S.sp.1-1/2.

Most intriguingly, we isolated a sequence from sporocysts of size class 4 that showed a high degree of similarity (98%) with the 18S rDNA of *S*. *nesbitti* from a muscle biopsy of a patient from Pangkor island in Malaysia (Fig 1) and another closely related human sample from Pangkor [9]. This similarity value is within the range of sequence variability observed among isolates of *S*. *nesbitti* from humans, macaques, and snakes (98%-100%), which we determined by pair wise BLAST comparison of *S*. *nesbitti* sequences (human: HF544323, HF544324, JX426074, JX661499; macaques: HM021725, HM021724; snakes: KC878476, KC878478, KC878479). Based on the high degree of sequence similarity with *S*. *nesbitti*-specific 18S rDNA and the results of our phylogenetic analysis, we address this isolate and the corresponding sporocysts (Fig 2) from the Australian scrub python as *Sarcocystis* cf. *nesbitti*, awaiting further characterization as discussed below. Although we could only retrieve sequences of *S*. *nesbitti* of one sample (sample 18) from class 4 sporocysts, this size class and morphology was observed three times among the 23 samples from scrub pythons (Table 2), suggesting that prevalence of infection may have been up to 13%.

When we employed Minimum Evolution (ME) and Maximum Likelihood (ML) algorithms for phylogeny reconstruction, all resulting trees consistently showed two monophyletic clades containing the bulk of the snake-host *Sarcocystis* spp.: here indicated as S1 and S2 and exemplified by an ME tree (Fig 1). While in this example clades S1 and S2 shared a common ancestor, one has to note that in some of our analyses, regardless of the substitution model or algorithm for phylogeny reconstruction, the *Sarcocystis* spp. of ruminants (as intermediate hosts) became a direct sister group of S2, which rendered the snake-host *Sarcocystis* spp. paraphyletic. It appeared that this split occurred more frequently once additional taxa were included in the tree. However, branch support for this scenario remained relatively weak. Isolates of *S*. *nesbitti*, including the new sequence from Australia (KY513626), formed a well-supported sub-clade of S2, which also contained *Sarcocystis* spp. from African, Asian, and American vipers and a colubrid snake. In contrast, clade S1 contained *Sarcocystis* spp. of colubrid and pythonid snakes distributed in Europe and or Asia, in particular those associated with the reticulated python (*S*. *singaporensis*, *S*. *zamani*) and the green tree python (KC201639, KC201640). Hence, considering that *Sarcocystis* sp.2 from the Australian spotted python clustered with taxa of branch C (Fig 1), our phylogenetic results indicated the existence of three snake-host lineages.

## Discussion

### Distribution range of *S*. *singaporensis*

Our results on the prevalence of *Sarcocystis* infection among pythons and molecular characterization indicate that *S*. *singaporensis* is widely distributed in the Indo-Australian Archipelago [30], probably also being a frequent coccidian parasite of the green tree python (*Morelia viridis*) in New Guinea. Although our own molecular analysis of the samples from green tree pythons was still ongoing at the time of writing and could not be included here, More et al. [31] isolated an 18S rDNA sequence of *Sarcocystis* from this snake that showed 97% sequence identity with *S*. *singaporensis*. Whether or not *S*. *singaporensis* is also present in Australia could not be determined by our study, since molecular sampling did not yield a *S*. *singaporensis*-specific sequence despite sporocysts of appropriate size (class 1) being present. Considering that half the pythons in the study area were infected with *Sarcocystis* spp., and given that *S*. *singaporensis* can reach high infection rates in its definitive host (see Table 2 and [27]), it is unlikely that our sample sizes were too small to detect an infection. It is more likely that, if present, *S*. *singaporensis* uses different snake-hosts in Australia—possibly the green tree python that is also native there [32], or carpet pythons (*Morelia spilota*). Previous work has shown at the light microscopical level that infection of laboratory rats using sporocysts isolated from the carpet python resulted in tissue cysts that resembled *S*. *singaporensis* [33]. Furthermore, examination of *Sarcocystis*-infected muscle tissue of Australian bush rats (*Rattus fuscipes*) revealed the typical ultrastructure of sarcocysts of *S*. *singaporensis* [34]. Focusing future research on these hosts should help clarify the status of *S*. *singaporensis* in Australia. These tests should also examine if isolates from New Guinea and Australia are infective for reticulated pythons in Southeast Asia. In the other direction, patent infections could be successfully induced in the black-headed python from Australia by feeding rats infected with *S*. *singaporensis* from Thailand [8].

### New *Sarcocystis* spp. and host associations in Australian pythons

The discovery of a *Sarcocystis* species (*Sarcocystis* sp.1) in the Australian scrub python that is closely related to *S*. *zamani* is interesting, because this suggests that *S*. *zamani* is present in Australia and uses a different snake-host there. This is not surprising, since muscle cysts that resemble *S*. *zamani* have been described from potential intermediate hosts like Australian bush rats [34]. Furthermore, the recent publication of a *Sarcocystis* 18S rDNA sequence from sporocysts in green tree pythons (presumably of Papua New Guinean origin; [31]) that clustered with *S*. *zamani* in our phylogenetic analyses (Fig 1; KC201639) renders it plausible that *S*. *zamani* also occurs in New Guinea, linking Southeast Asia with Australia. A sequence variation of up to 2% divergence that was present among our samples of *Sarcocystis* sp.1 indicates a higher degree of variability than the variation observed among isolates of *S*. *singaporensis* throughout large parts of its distribution range. However, this is under the restriction that we used smaller fragments of 18S rDNA for alignment of sequences of *S*. *singaporensis* compared to those of *Sarcocystis* sp.1. Our observations are in line with phylogenetic analyses of the 28S rRNA gene of *S*. *zamani*, where two genetically distinct sub-populations of this species were described [29]. However, that study did not link the genetic findings with histological or ultrastructural evidence for presence of *S*. *zamani*, so it remains unclear whether or not genetic variability correlated with morphological disparities. The existence of two sub-populations of *S*. *zamani* sporocysts from Southeast Asia that were different in size [26,27], and our discovery of even larger sporocysts of a putative *S*. *zamani* in Australia, also suggest a relatively high degree of phenotypic variability. Taken together, these uncertainties prevent us from confirming the presence of *S*. *zamani* in Australia.

*Sarcocystis* sp. 2 was clearly distinct to the other snake-host *Sarcocystis* spp. of this study (taxa of clades S1 and S2). The 18S rRNA gene sequence of *Sarcocystis* sp. 2 clustered with a group of *Sarcocystis* spp. that includes a diverse range of definitive hosts (branch C of Fig 1), which is regarded as poorly sampled [24]. Evidence is now accumulating that this group of taxa not only contains *S*. *lacertae*, which cycles between the colubrid snake *Coronella austriaca* as definitive host and the common wall lizard *Podarcis muralis* as intermediate host [28], but also other snake-host *Sarcocystis* spp.; for instance those that use neotropical tree boas of the genus *Corallus* as definitive hosts [15]. This implies the existence of a third lineage of snake-host *Sarcocystis* spp. that is distinct from the taxa of clades S1 and S2. We suggest that the hypothesis for a single lineage of snake-specific *Sarcocystis* spp. that has co-evolved with their definitive hosts is in need of revision [35,36].

Because most of the Apicomplexan protozoa are only known from sequences of the 18S rRNA gene [37]–and this is particularly true for the snake-host *Sarcocystis* spp.–our reconstruction of the tree of different taxa reflects the phylogeny of the gene rather than the species involved. To construct a species phylogeny, multiple gene sequences, best coupled with phenotypic characters should be analysed [37]. To that end, additional genes should be sequenced, such as the mitochondrial cytochrome oxidase 1 (*cox1*) of the new *Sarcocystis* isolates examined here. This gene has been found useful for discriminating closely related *Sarcocystis* spp. [38,39]. Additionally, such investigations should be complemented with cross-infection experiments as outlined for *S*. *singaporensis* to clarify hosts and distributions beyond Australia.

### The definitive host of *S*. *nesbitti*

We report here for the first time the potential presence of *S*. *nesbitti* in Australia. We provide molecular and morphological evidence for the stage (sporocyst containing sporozoites) developing in Australian scrub pythons. This discovery is remarkable in several ways. First, it confirms that a snake is indeed the definitive host—as was suggested after phylogenetic analysis of 18S rDNA from sarcocysts of infected monkeys [13]. However, we did not expect *S*. *nesbitti’*s distribution to extend to Australia, because non-human primates that were identified as intermediate hosts do not occur there [13, 40–42]. Therefore, if this parasite is present in Australia (in absence of primates), this immediately leads to a quest for the potential intermediate hosts involved in its life cycle. Australian scrub pythons prefer rainforest habitats, where they prey on birds and mammals, including rodents and wallabies (D. Natusch unpubl. observations; [43]). Therefore, we postulate that *S*. *nesbitti* might exhibit an ‘ordinary’ snake-rodent life cycle, possibly with a lower degree of specificity for the intermediate host (which would account for its presence in primates and humans). Another line of support for this assumption comes from the phylogenetic analysis: If the host-parasite relationships of the *Sarcocystis* species with snake-rodent life cycle reflect the true evolutionary relationships among these taxa [24], then the prediction that *S*. *nesbitti* may use rodents as intermediate host is a relatively robust one. All taxa of clades S1 and S2, except *S*. *nesbitti*, are either known to develop in rodents or have been isolated from snakes that are known rodent-eaters. Again, reservations regarding species identification based on a single gene sequence also apply here. Infection experiments with the Australian isolate are required to determine its host range and morphological traits, including further molecular characterization.

Based on previous research, we expected the reticulated python of Southeast Asia to be *S*. *nesbitti*’s definitive host [14]. However, we did not find *S*. *nesbitti* in this snake, despite extensive sampling of animals and locations in the region. Considering that we possibly detected three *S*. *nesbitti* infections in 23 scrub pythons (while acknowledging that only one was confirmed by molecular analysis), we would have expected to find at least one or two infected reticulated pythons among our samples if this snake species was a suitable definitive host. Therefore, we do not believe that the reticulated python is a suitable definitive host for *S*. *nesbitti*, although we cannot rule out this possibility until infection experiments can provide a clear answer.

Which snake species could be the definitive host of *S*. *nesbitti* in Southeast Asia? Our finding of *S*. *nesbitti* in *Simalia amethistina* suggests a close relative to that species as a likely candidate, perhaps among the Afro-Asian pythons (e.g., *Python bivittatus* or *P*. *brongersmai*; [44]). However, our phylogenetic analysis appears to contradict that hypothesis, since *S*. *nesbitti* did not cluster with *S*. *singaporensis* or *S*. *zamani*, which are both associated with pythons. Instead, it grouped with taxa of clade S2, which included *Sarcocystis* spp. of colubrid and viperid snakes. This could imply that *S*. *nesbitti* uses snakes of different families as definitive hosts. For example, *S*. *muriviperae* appears to develop in colubrid as well as viperid snakes [45]. On the other hand, clade S2 may still be under-sampled; in that case additional sampling could reveal more python hosts in this group.

With regard to the outbreaks on Tioman Island [10,46] we are reluctant to accept a reticulated python-long-tailed macaque life cycle for *S*. *nesbitti* as hypothesized by Shahari and co-workers [47]. Adult reticulated pythons do prey on long-tailed macaques among other larger mammals, but only once they have attained a certain size (3–4 m total length in South Sumatra; [48,49]); smaller pythons mainly feed on rats [49]. Thus, even if macaques occasionally form part of their diet on Tioman, the volume of infectious sporocysts released into the environment would not be sufficient to explain the apparently widespread contamination of village water sources [10]. In order to find the source of infection, we suggest future investigators search the headwaters of streams flowing into areas where significant human infections occurred [10,50], and sample snake species found nearby to these water bodies. Trapping and examination of rodents in these same areas should also be undertaken.

In conclusion, the potential presence of *S*. *nesbitti* in the Australian scrub python extends the known range of this pathogen significantly, and warrants further investigations into its relationship with Malaysian isolates and disease-causing properties. If confirmed, this would have implications for human health in the region. Australian authorities and medical personnel should be made aware of the parasite’s potential presence and further sampling should be undertaken in more populated parts of the country.

## References

[1] Odening K. The present state of species-systematics in *Sarcocystis* Lankester, 1882 (Protista, Sporozoa, Coccidia). Syst Parasitol 1998;41:209–33.

[2] Fayer R, Esposito DH, Dubey JP. Human infections with *Sarcocystis* species. Clin Microbiol Rev. 2015;28(2):295–311. doi: 10.1128/CMR.00113-14 25715644 10.1128/CMR.00113-14 PMC4402950

[3] Dubey JP, Calero-Bernal R, Rosenthal BM, Speer CA, Fayer R. Sarcocystosis of Animals and Humans. Boca Raton: CRC Press, Taylor & Francis Group; 2016.

[4] Jäkel T, Khoprasert Y, Promkerd P, Hongnark S. An experimental field study to assess the effectiveness of bait containing the parasitic protozoan *Sarcocystis singaporensis* for protecting rice crops against rodent damage. Crop Prot. 2006;25:773–80.

[5] Zaman V, Colley FC. Light and electron microscopic observations of the life cycle of *Sarcocystis orientalis* sp. n. in the rat (*Rattus norvegicus*) and the Malaysian reticulated python (*Python reticulatus*). Z Parasitenkd. 1975;47(3):169–85. 810990 10.1007/BF00418200

[6] Zaman V. Host range of *Sarcocystis orientalis*. Southeast Asian J Trop Med Public Health. 1976;(1):112 829174

[7] Beaver PC, Maleckar JR. *Sarcocystis singaporensis* Zaman and Colley, (1975) 1976, *Sarcocystis villivillosi* sp. n., and *Sarcocystis zamani* sp. n.: development, morphology, and persistence in the laboratory rat, *Rattus norvegicus*. J Parasitol. 1981;67(2):241–56. 6787185

[8] Häfner U, Frank W. Host specificity and host range of the genus *Sarcocystis* in three snake-rodent life cycles. Zentralbl Bakteriol Mikrobiol Hyg A. 1984;256(3):296–9. 6428079

[9] AbuBakar S, Teoh BT, Sam SS, Chang LY, Johari J, Hooi PS, et al Outbreak of human infection with *Sarcocystis nesbitti*, Malaysia, 2012. Emerg Infect Dis. 2013;19(12):1989–91. doi: 10.3201/eid1912.120530 24274071 10.3201/eid1912.120530 PMC3840867

[10] Esposito DH, Stich A, Epelboin L, Malvy D, Han PV, Bottieau E, et al Acute muscular sarcocystosis: an international investigation among ill travelers returning from Tioman Island, Malaysia, 2011–2012. Clin Infect Dis. 2014;59(10):1401–10. doi: 10.1093/cid/ciu622 25091309 10.1093/cid/ciu622 PMC4624310

[11] Italiano CM, Wong KT, AbuBakar S, Lau YL, Ramli N, Syed Omar SF, et al *Sarcocystis nesbitti* causes acute, relapsing febrile myositis with a high attack rate: description of a large outbreak of muscular sarcocystosis in Pangkor Island, Malaysia, 2012. PLoS Negl Trop Dis. 2014;8(5):e2876 doi: 10.1371/journal.pntd.0002876 24854350 10.1371/journal.pntd.0002876 PMC4031117

[12] Arness MK, Brown JD, Dubey JP, Neafie RC, Granstrom DE. An outbreak of acute eosinophilic myositis attributed to human *Sarcocystis* parasitism. Am J Trop Med Hyg. 1999;61(4):548–53. 10548287 10.4269/ajtmh.1999.61.548

[13] Tian M, Chen Y, Wu L, Rosenthal BM, Liu X, He Y, et al Phylogenetic analysis of *Sarcocystis nesbitti* (Coccidia: Sarcocystidae) suggests a snake as its probable definitive host. Vet Parasitol. 2012;183(3–4):373–6. doi: 10.1016/j.vetpar.2011.07.034 21852042 10.1016/j.vetpar.2011.07.034

[14] Lau YL, Chang PY, Subramaniam V, Ng YH, Mahmud R, Ahmad AF, et al Genetic assemblage of *Sarcocystis* spp. in Malaysian snakes. Parasit Vectors. 2013;6(1):257 doi: 10.1186/1756-3305-6-257 24010903 10.1186/1756-3305-6-257 PMC3847168

[15] Abe N, Matsubara K, Tamukai K, Miwa Y, Takami K. Molecular evidence of *Sarcocystis* species in captive snakes in Japan. Parasitol Res. 2015;114(8):3175–9. doi: 10.1007/s00436-015-4564-2 26044884 10.1007/s00436-015-4564-2

[16] Lau YL, Chang PY, Tan CT, Fong MY, Mahmud R, Wong KT. *Sarcocystis nesbitti* infection in human skeletal muscle: possible transmission from snakes. Am J Trop Med Hyg. 2014;90(2):361–4. doi: 10.4269/ajtmh.12-0678 24420776 10.4269/ajtmh.12-0678 PMC3919249

[17] Greiner EC, Mader DR. Parasitology In: Mader DR, editor. Reptile Medicine and Surgery. St. Louis, Missouri, USA: Saunders Elsevier; 2006 p. 343–64.

[18] Furlan E, Gleeson DM. Environmental DNA detection of redfin perch, *Perca fluviatilis*. Conserv Genet Resour. 2016 doi: 10.1007/s12686-016-0516-0

[19] Holmdahl OJ, Morrison DA, Ellis JT, Huong LT. Evolution of ruminant *Sarcocystis* (Sporozoa) parasites based on small subunit rDNA sequences. Mol Phylogenet Evol. 1999;11(1):27–37. doi: 10.1006/mpev.1998.0556 10082608 10.1006/mpev.1998.0556

[20] Slapeta JR, Kyselova I, Richardson AO, Modry D, Lukes J. Phylogeny and sequence variability of the *Sarcocystis singaporensis* Zaman and Colley, (1975) 1976 ssrDNA. Parasitol Res. 2002;88(9):810–5. doi: 10.1007/s00436-002-0657-9 12172812 10.1007/s00436-002-0657-9

[21] Clark K, Karsch-Mizrachi I, Lipman DL, Ostell J, Sayers EW. GenBank Nucleic Acids Res. 2016;44:D67–D72. doi: 10.1093/nar/gkv1276 26590407 10.1093/nar/gkv1276 PMC4702903

[22] Moretti S, Wilm A, Higgins DG, Xenarios I, Notredame C. R-Coffee: a web server for accurately aligning noncoding RNA sequences. Nucleic Acids Res. 2008;36 (Web Server issue)(W10-3). doi: 10.1093/nar/gkn278 18483080 10.1093/nar/gkn278 PMC2447777

[23] Darriba D, Taboada GL, Doallo R, Posada D. jModelTest 2: more models, new heuristics and parallel computing. Nature Methods. 2012;9(8):772. 10.1038/nmeth.2109 PMC4594756 22847109

[24] Morrison DA, Bornstein S, Thebo P, Wernery U, Kinne J, Mattsson JG. The current status of the small subunit rRNA phylogeny of the coccidia (Sporozoa). Int J Parasitol. 2004;34(4):501–14. doi: 10.1016/j.ijpara.2003.11.006 15013740 10.1016/j.ijpara.2003.11.006

[25] Tamura K, Stecher G, Peterson D, Filipski A, Kumar S. MEGA6: Molecular Evolutionary Genetics Analysis version 6.0. Mol Biol Evol. 2013;30:2725–9. doi: 10.1093/molbev/mst197 24132122 10.1093/molbev/mst197 PMC3840312

[26] Häfner U. Zystenbildende Coccidien mit Nager/Schlange-Zyklen unter besonderer Beruecksichtigung der Wirtsspezifitaet der Gattung *Sarcocystis* [in German]. Stuttgart-Hohenheim: Dissertation, University of Hohenheim; 1987.

[27] Jäkel T, Khoprasert Y, Sorger I, Kliemt D, Seehabutr V, Suasa-ard K, et al Sarcosporidiasis in rodents from Thailand. J Wildl Dis. 1997;33(4):860–7. doi: 10.7589/0090-3558-33.4.860 9391972 10.7589/0090-3558-33.4.860

[28] Volf J, Modrý D, Koudela B, Šlapeta JR. Discovery of the life cycle of *Sarcocystis lacertae* Babudieri, 1932 (Apicomplexa: Sarcocystidae), with a species redescription. Folia Parasitol (Praha). 1999;46:257–62.

[29] Watthanakaiwan V, Wajjwalku W, editors. Biodiversity of Sarcocystis zamani based on 28S rRNA gene in rodent pest Bandicota and Rattus species in Thailand. Proceedings of The 53rd Kasetsart University Annual Conference; 2015; Kasetsart University, Bangkok, Thailand.

[30] Lohman DL, de Bruyn M, Page T, von Rintelen K, Hall R, Ng PKL, et al Biogeography of the Indo-Australian Archipelago. Rev Ecol Evol Syst. 2011;42:205–26.

[31] More G, Pantchev N, Herrmann DC, Vrhovec MG, Ofner S, Conraths FJ, et al Molecular identification of *Sarcocystis* spp. helped to define the origin of green pythons (*Morelia viridis*) confiscated in Germany. Parasitology. 2013;141(5):646–51. doi: 10.1017/S0031182013001960 24476633 10.1017/S0031182013001960

[32] Rawlings LH, Donnellana SC. Phylogeographic analysis of the green python, *Morelia viridis*, reveals cryptic diversity. Mol Phylogenet Evol. 2003;27:36–44. 12679069 10.1016/s1055-7903(02)00396-2

[33] Rzepczyk CM. Evidence of a rat-snake life cycle for *Sarcocystis*. Int J Parasitol. 1974;4(4):447–9. 4217324 10.1016/0020-7519(74)90058-7

[34] Rzepczyk C, Scholtyseck E. Light and electron microscope studies on the *Sarcocystis* of *Rattus fuscipes*, an Australian rat. Z Parasitenkd. 1976;50(2):137–50. 822623 10.1007/BF00380518

[35] Verma SK, Lindsay DS, Rosenthal BM, Dubey JP. Ancient, globally distributed lineage of *Sarcocystis* from sporocysts of the Eastern rat snake (*Pantherophis alleghaniensis*) and its relation to neurological sequalae in intermediate hosts. Parasitol Res. 2016 doi: 10.1007/s00436-016-5086-2 27130321 10.1007/s00436-016-5086-2

[36] Slapeta JR, Modry D, Votypka J, Jirku M, Lukes J, Koudela B. Evolutionary relationships among cyst-forming coccidia *Sarcocystis* spp. (Alveolata: Apicomplexa: Coccidea) in endemic African tree vipers and perspective for evolution of heteroxenous life cycle. Mol Phylogenet Evol. 2003;27(3):464–75. 12742751 10.1016/s1055-7903(03)00018-6

[37] Morrison DA. Evolution of the Apicomplexa: where are we now? Trends in Parasitology. 2009;25:375–82. doi: 10.1016/j.pt.2009.05.010 19635681 10.1016/j.pt.2009.05.010

[38] Gjerde B. Phylogenetic relationships among *Sarcocystis* species in cervids, cattle and sheep inferred from the mitochondrial cytochrome c oxidase subunit I gene. Int J Parasitol. 2013;43(7):579–91. doi: 10.1016/j.ijpara.2013.02.004 23542092 10.1016/j.ijpara.2013.02.004

[39] Poulsen CS, Stensvold CR. Current Status of Epidemiology and Diagnosis of Human Sarcocystosis. J Clin Microbiol. 2014;52:3524–30. doi: 10.1128/JCM.00955-14 24759707 10.1128/JCM.00955-14 PMC4187749

[40] Mandour AM. *Sarcocystis nesbitti* n. sp. from the rhesus monkey. J Protozool. 1969;16(2):353–4. 4978784 10.1111/j.1550-7408.1969.tb02281.x

[41] Yang ZQ, Wei CG, Zen JS, Song JL, Zuo YX, He YS, et al A taxonomic re-appraisal of *Sarcocystis nesbitti* (Protozoa: Sarcocystidae) from the monkey *Macaca fascicularis* in Yunnan, PR China. Parasitol Int. 2005;54(1):75–81. doi: 10.1016/j.parint.2004.12.004 15710555 10.1016/j.parint.2004.12.004

[42] Duff A, Lawson A. Mammals of the World: A Checklist. New Haven, Connecticut, USA: Yale University Press; 2004. 312 pp.

[43] Shine R, Slip DJ. Biological aspects of the adaptive radiation of Australasian pythons (Serpentes: Boidae). Herpetologica. 1990;46:283–90.

[44] Rawlings LH, Rabosky DL, Donnellan SC, Hutchinson MN. Python phylogenetics: inference from morphology and mitochondrial DNA. Biol J Linnean Soc. 2008;93:603–19.

[45] Paperna I, Finkelman S. Ultrastructural study of *Sarcocystis muriviperae* development in the intestine of its snake hosts. Folia Parasitol (Praha). 1996;41(1):13–9. 8682406

[46] Tappe D, Stich A, Langeheinecke A, von Sonnenburg F, Muntau B, Schäfer J, et al Suspected new wave of muscular sarcocystosis in travellers returning from Tioman Island, Malaysia, May 2014. Euro Surveill. 2014;19(21):pli = 20816. 10.2807/1560-7917.es2014.19.21.20816 24906376

[47] Shahari S, Tengku-Idris TIN, Fong MY, Lau YL. Molecular evidence of *Sarcocystis nesbitti* in water samples of Tioman Island, Malaysia. Parasit Vectors. 2016;9:598 doi: 10.1186/s13071-016-1883-9 27881179 10.1186/s13071-016-1883-9 PMC5120450

[48] Headland TN, Greene HW. Hunter-gatherers and other primates as prey, predators, and competitors of snakes. PNAS. 2011;108(52):E1470–E4. doi: 10.1073/pnas.1115116108 22160702 10.1073/pnas.1115116108 PMC3248510

[49] Shine R, Harlow PS, Keogh JS, Boeadi NI. The influence of sex and body size on food habits of a giant tropical snake, *Python reticulatus*. Funct Ecol. 1998;12:248–58.

[50] Daulay HB, Rahman NA, Kassim AHM, Nasir KAM. Study on water resources in Tioman Island. Jurnal Teknologi. 2001;34:51–64.

